# Anti-coagulation Drug Warfarin Contributes to Severe Adverse Outcomes in Prolonged Unsupervised Use: A Double-edged Sword

**DOI:** 10.7759/cureus.3347

**Published:** 2018-09-22

**Authors:** Christ Ordookhanian, Sean W Kaloostian, Paul E Kaloostian

**Affiliations:** 1 Neurological Surgery, University of California Riverside School of Medicine, Riverside, USA; 2 Neurological Surgery, Haider Spine Center, Riverside, USA

**Keywords:** warfarin, anti-coagulation therapy, coumadin, quadriplegia, methylprednisolone, spinal stenosis, congestive heart failure, adverse effect, pan neuroaxis

## Abstract

Anti-coagulation medications are widely used in clinical practice, especially in the United States as cardiac-related emergencies are on the rise. An overarching caveat in using anti-coagulation drugs as a long-term treatment regimen, such as warfarin, is that patients are closely monitored by their primary care provider. Routine monthly laboratory examinations are strictly required to assess the international normalized ratio (INR) which measures prothrombin time (PT), providing insight into how long it takes for blood to clot. A 58-year-old Caucasian male presented with non-traumatic acute quadriplegia with cord compression starting at the 3rd cervical (C3) level and continuing throughout the entire spinal cord. Radiological studies revealed severe cervical stenosis from C3 to C7 resulting from a posterior spinal mass, thoracic and lumbar spine studies revealed a compressive posterior acute epidural hematoma (EDH) compressing the thoracic spinal cord from T6 to T10 with signal change, as well as a L4-S1 posterior acute epidural hematoma compressive of the cauda equina. Upon detailed review of our patient’s history it became evident that our patient had been on a prolonged and unsupervised warfarin (Coumadin) regimen for congestive heart failure without primary care follow-up or routine laboratory testing. Our patient was a prime surgical candidate for an immediate two-stage decompression, hematoma evacuation and instrumentated fusion surgery. Post-operatively, our patient regained leg strength to 2/5 and arm strength to 4+/5.

## Introduction

Warfarin (branded coumadin) is a prescription anticoagulant medication generally prescribed to patients that are diagnosed with congestive heart failure (CHF). Warfarin inhibits the vitamin K epoxide reductase enzyme that reactivates vitamin K1 which reduces the body's coagulation ability. Vitamin K epoxide reductase enzyme inhibition therefore inadequately activates vitamin K dependent coagulation factors, subsequently achieving the goal of anticoagulation. Proper usage of warfarin involves the close monitoring of patients' international normalized ratio (INR) to maintain optimal range of 2.0–3.0. Adequate monitoring of patients on prolonged anti-coagulation involves the frequent testing of INR and prothrombin. Warfarin possesses many side effects with primary concern being the increased risk of bleeding. In rare cases, approximately 1–3% of warfarin using patients will experience severe bleeding which will result in intracerebral haemorrhage or epidural hematoma formation within the spinal cord [1]. When the INR, which measures prothrombin time (PT) and reports on how long it takes for blood to clot, is out of range the risk of bleeding significantly increases for patients with INR > 4.5; the risk of bleeding is extremely severe and requires immediate medical intervention [2, 3]. The INR level within the plasma is an essential test that must be conducted especially in patients that are prone to bleeding, or have had previous haemorrhaging experiences. Our patient at initial presentation had an INR of 5.0 which is highly indicative for the need of medical intervention and predisposes the patient to spontaneous haemorrhage within the body. The patient's past medical history was significant for a long-term warfarin regimen without proper regulation, testing and deniance of primary care physician guidance. The impact of this case provides for a valuable teaching point for both physicians and patients. As a physician we have the ultimate responsibility for guiding and providing our patients with the most mutually beneficial prognosis for remediation of any medical pathology. As a patient, it is imperative for one to take on an interactive role in his healthcare by following instructions from medical professionals, especially when on a regimen of high-risk medication such as warfarin. For the medical community this case presents a rare and unique presentation where the small 1–3% chance of severe side effect occurrence became a reality and significantly impacted the patient’s quality of life. This case not only serves as a reminder of an important clinical lesson for primary care physicians to follow up routinely with patients taking anticoagulant medications, but also incorporates a lesson that can be learned from a medical error that was made on the part of all physicians treating our patient. Most impactfully to the emergency medicine and surgical community, the occurrence of a pan neuroaxis epidural hematoma is extremely rare, unique and novel to medical literature. Our patient’s epidural hematoma comprises the pan neuroaxis and necessitated for a two-step surgical approach for complete eradication.

## Case presentation

**Figure 1:**
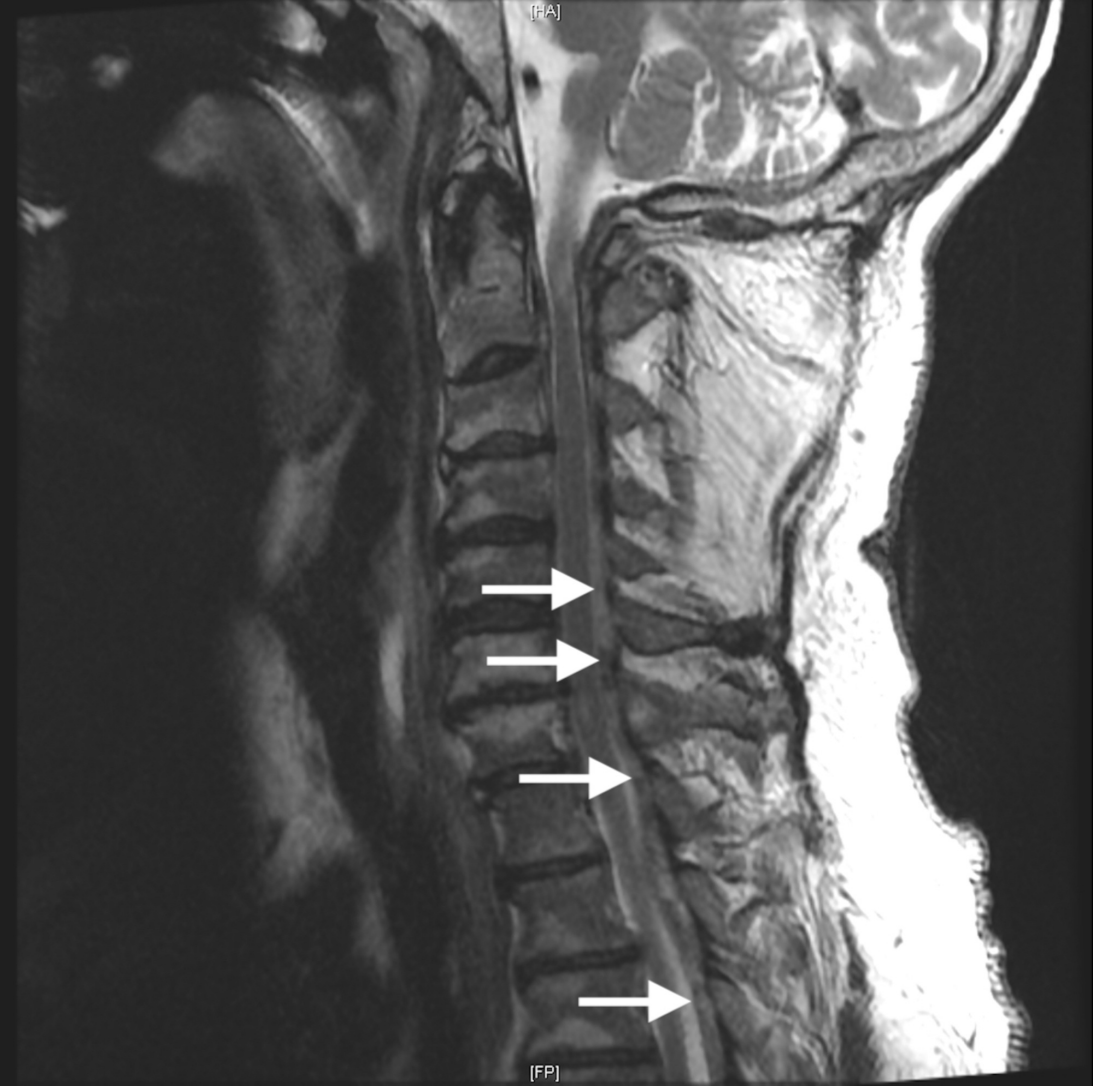
Sagittal T2 magnetic resonance imaging (MRI) of the cervical spine, with C3 to C7 involved in the pathology described in this report. Sagittal T2 MRI presenting the patient's cervical spine where denoted descending arrows highlight the presentation of the C3 to C7 hematoma, respectively.

Our patient, a 58-year-old Caucasian male, presented to the emergency department with the acute onset of quadriplegia extending from C5 throughout the rest of the pan neuroaxis while awakening from bed the morning of presentation. Upon initial physical examination there were no signs of trauma noted that were significant to the presenting symptoms, additionally, patchy sensation was noted in the upper and lower extremities with clonus in the legs only and hyperreflexia in both arms and legs. Initially, our patient was administered one intravenous dose of methylprednisolone and allowed to enter a state of permissive hypertension which improved our patient's arm strength bilaterally to 2/5, but had no positive effects on the lower extremity paraplegia. As a result of minimal improvement, immediate imaging of the cervical spine was ordered and highlighted a severe cervical stenosis from C3 to C7, as seen in Figure 1, due to extra-axial posterior compressive spinal mass with cord signal change.

**Figure 2:**
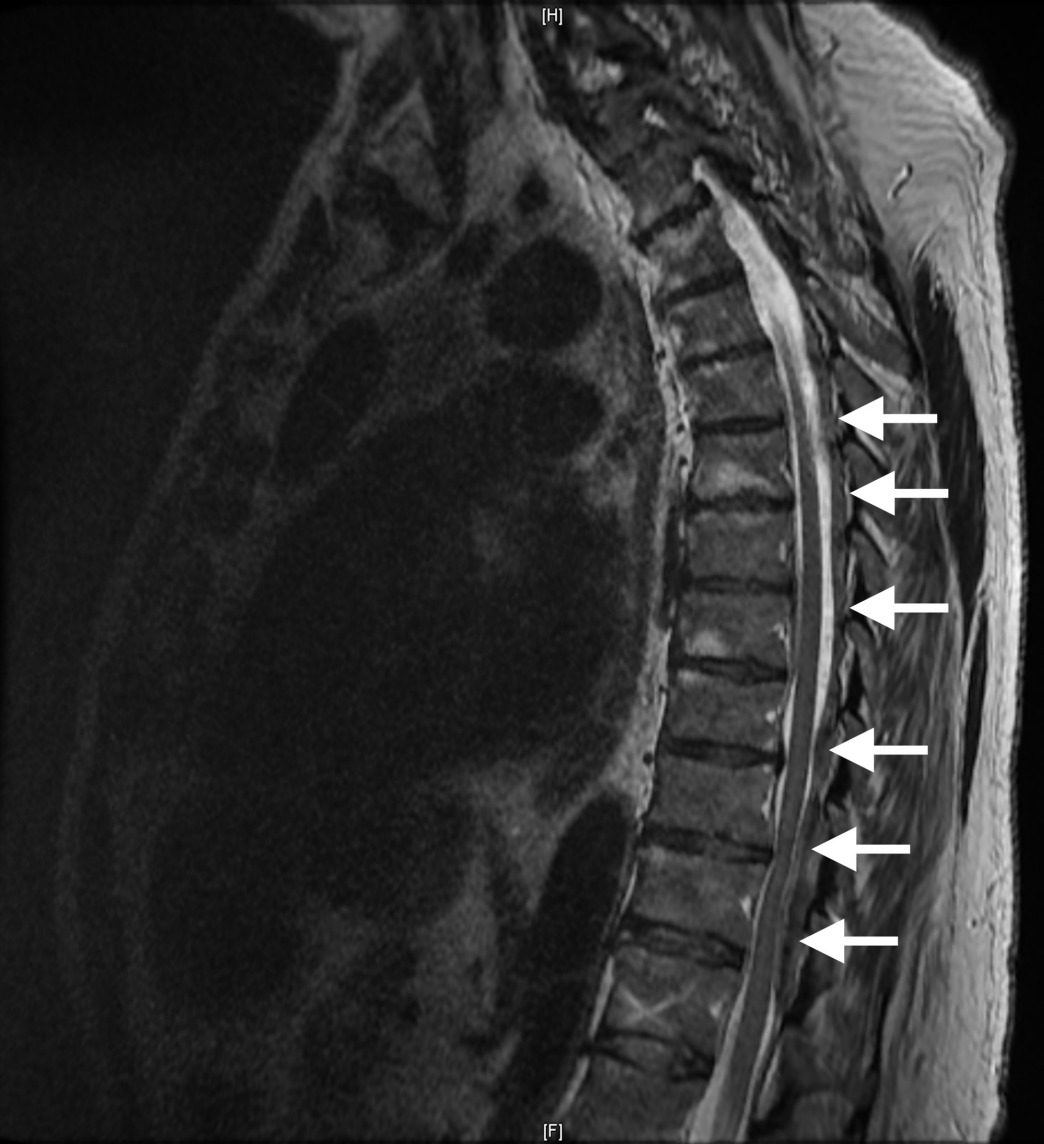
Sagittal T2 magnetic resonance imaging (MRI) of the thoracic spine, with T6 to T10 involved in the pathology described in this report. Sagittal T2 MRI presenting a severe and wide-range hematoma of the thoracic spine where the denoted arrows highlight the densely populated and highly constrictive pathology.

**Figure 3:**
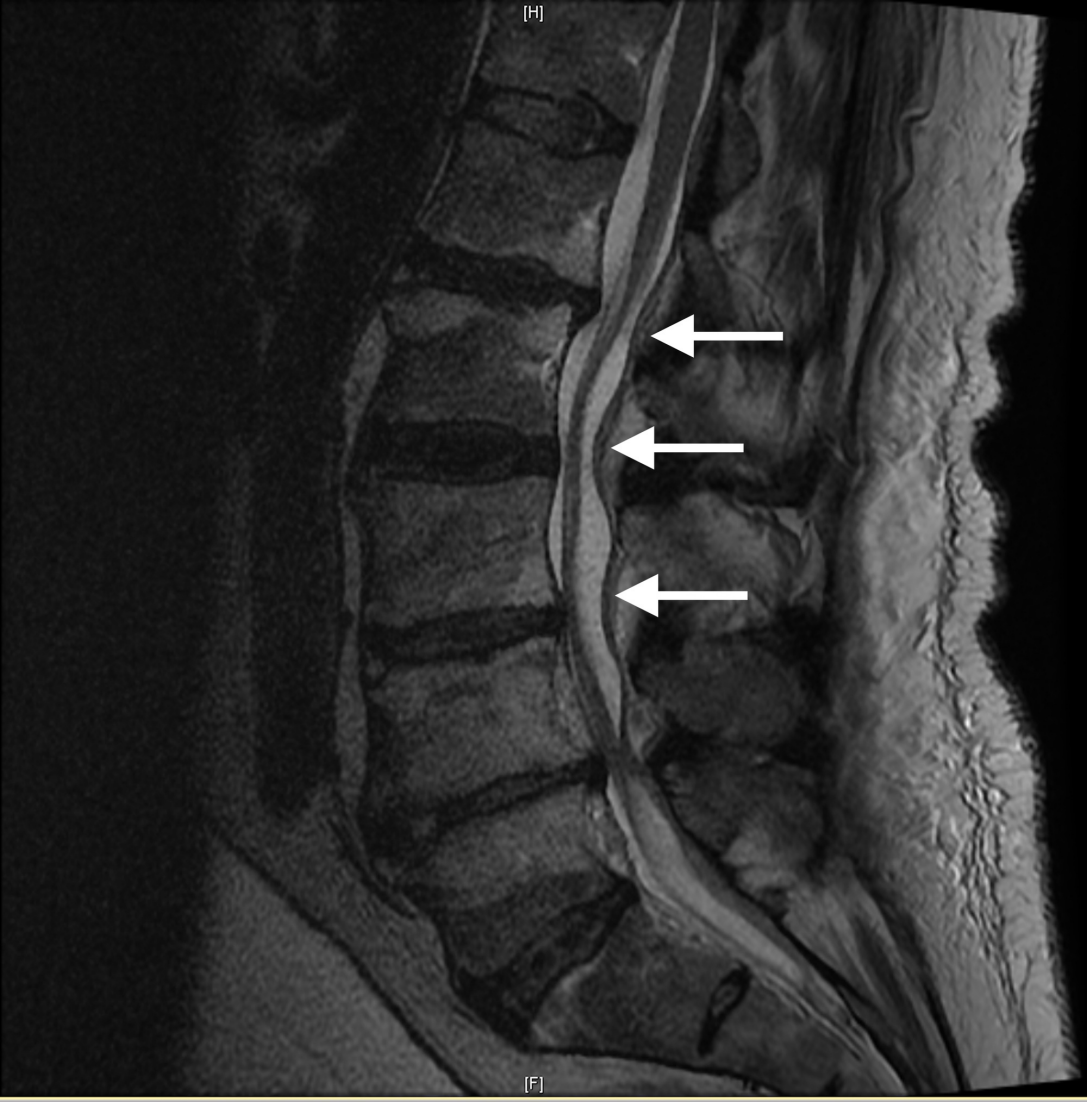
Sagittal T2 magnetic resonance imaging (MRI) of the lumbar-sacral spine, with L4 to S1 involved in the pathology described in this report. Sagittal T2 MRI of the lumbar-sacral spine region where the pan-neuroaxis hematoma concludes its dominant presentation. The arrows in descending order highlight the coverage of the hematoma from the L4 to the S1 spine region, respectively.

Additional imaging of the brain was completed as a precautionary measure and was normal. Upon obtaining full patient history and medical records from nearby hospitals, it was found that our patient was on a prolonged regimen of warfarin for many years due to a prior diagnosis of congestive heart failure without regulation and regular INR/prothrombin evaluation. Our patient also did not follow up with his primary care physician throughout the duration of the warfarin regimen. At presentation, our patient's INR was found to be 5.0 necessitating the prompt correction with intravenous injection of fresh frozen plasma (FFP) and vitamin K. Additional magnetic resonance imaging (MRI) of the thoracic and lumbar spine revealed large compressive acute epidural hematoma posteriorly compressing the thoracic spinal cord from T6 to T10, visualized in Figure 2, with cord signal change, as well as L4-S1 posterior acute epidural hematoma compressive of the cauda equine, visualized in Figure 3.

Upon a complete review of the patient history, presenting symptoms, and radiological imaging results our patient was deemed a prime surgical candidate that would significantly improve both motor function, sensation, and ultimate restoration of our patient's quality of life. Our patient was emergently taken to the operating room for stage 1 of a two-part surgical procedure. Stage 1 involved a posterior T3 to L1 decompression, epidural hematoma evacuation and instrumented fusion. Three days later, our patient was taken to the operating room again for stage 2 of the planed procedure which involved a posterior cervicothoracic C2 to T2 decompression and instrumented fusion with evacuation of acute epidural hematoma.

## Discussion

Warfarin therapy has been widely utilized for nearly 70 years and continues to be one of the leading causes of medication-related adverse effects. Warfarin therapy produces astonishingly positive results if taken under the strict guidance of a professional healthcare provider. Over the last few years many research groups have begun to take novel strides in developing dosage algorithms that include genetic information for optimal patient outcomes [4]. To date, for patients with a history of congestive heart failure with a high probability of thromboembolism, the oral form and long-term use approved medications are aspirin and warfarin. However, as prescribing history has shown, warfarin is clearly the most popular choice for the prevention of thromboembolism as it is a remarkably effective drug [5]. As the population demand for anticoagulation drugs continues to increase, it is with no doubt that the demand for warfarin-based therapies will continue to increase. Thus, patients and physicians considering this therapy must adhere to strict management and regulation to ensure patient safety and prevention of adverse side effects. In the event where warfarin is problematic for the patient's prognosis, it is essential to administer 1–2 mg of vitamin K1 or K2 depending on if the patient has active bleeding and INR at the presentation. For patients that have no bleeding and an INR above 4.5–5, administration of 1 mg vitamin K1 by mouth is recommended. For presenting cases with active bleeding, classification of bleeding into the following categories is crucial: minor, elevated without haemodynamic compromise, and life-threatening. For patients with minor active bleeding, administration of 2 mg vitamin K2 by mouth is recommended; in cases with elevated bleeding, administration of 1 mg vitamin K1 intravenously is recommended with consideration for fresh frozen plasma (FFP). In most severe cases where active bleeding is considered life-threatening, it is recommended to consult a hematologist for the potential administration of 5 mg vitamin K and prothrombin complex concentrate. Upon review of literature, a case similar to ours highlighted by Yabe et al. in 2012 shows that the occurrence of spontaneous spinal epidural hematomas as a result of long-term warfarin therapy is rare but does come with warning signs such as unexplained cervical/back pain or subsequent radicular discomfort [6]. In the case, an 80-year-old male patient developed spinal epidural hematoma involving the C5 to C7 region during a long-term warfarin therapy, for which surgical evacuation of the hematoma was sufficient to relieve pain and restore extremity strength [6]. Another case similar to ours was highlighted by Anwar et al. in 2010. Their 58-year-old male patient also presented with lack of extremity strength and eventual paraplegia which was proven through MRI studies to be resulting from a C4 to T2 spinal epidural hematoma and was also revealed through hematoma evacuation [7]. Both cases briefly discussed above represent a group of patients in a relatively similar age group presenting with similar symptoms like our patient and were reported in recent literature. Of the patients described above, the only appropriate surgical procedure was hematoma evacuation and subsequent conservative rehabilitative efforts. The spinal cord compression in the cases above was minimal and non-indicatory for instrumented fusion.

## Conclusions

The significance of our case from that cases published in literature today is that we present a case that is extremely rare in occurrence and reports on a case for which warfarin therapy leads to epidural hematoma comprising the pan neuroaxis, a novel addition to medical literature. Many cases of localized hematomas are present in literature today, however, our case demonstrates the rare pathology of a pan-axis epidural hematoma that occurred as a result of partial-iatrogenic/partial-noncompliance complications. Our patient’s pan neuroaxis warfarin-associated epidural hematoma comprised the cervical (C3 to C7), thoracic (T6 to T10), as well as lumbar and sacral (L4 to S1) regions of the spine. Additionally, our patient's presenting pathology was extremely severe and aggressive in nature, such as: spinal cord signal change was present, compression of multiple spinal cord regions causing limited ambulatory ability, and debilitating physical strength concerns. Our two-stage surgical procedure not only involved the evacuation of the multiple hematomas but also included spinal cord decompression along with instrumented spinal fusion.
